# *N*-Paranitrophénylhydrazono-α-(2-méthyl­benzimidazol-1-yl)glyoxylate d’éthyle

**DOI:** 10.1107/S1600536811022148

**Published:** 2011-06-11

**Authors:** Aicha Boudina, Abdesselam Baouid, Mohamed Driss, El Hassane Soumhi

**Affiliations:** a Équipe de Chimie des Hétérocycles et Valorisation des Éxtraits des Plantes, Faculté des Sciences-Semlalia, Université Cadi Ayyad, Boulevard Abdelkrim Khattabi, BP 2390, 40001 Marrakech, Maroc; b Laboratoire de Matériaux et Cristallochimie, Faculté des Sciences de Tunis, Université de Tunis El Manar, 2092 El Manar II Tunis, Tunisie; c Équipe de Chimie des Matériaux et de l’Environnement, FSTG-Marrakech, Université Cadi Ayyad, Boulevard Abdelkrim Khattabi, BP 549, Marrakech, Maroc

## Abstract

There are two independent mol­ecules in the asymmetric unit of the title compound {systematic name: ethyl 2-(2-methyl-1*H*-benzimidazol-1-yl)-2-[2-(4-nitro­phen­yl)hydrazinyl­idene]ethano­ate}, C_18_H_17_N_5_O_4_. Each mol­ecule and its inversion-related partner are linked by a pair of inter­molecular N—H⋯N hydrogen bonds, forming inversion dimers in the crystal structure.

## Littérature associée

Pour le contexte général des derivées des benzodiazépines et benzotriazépines, voir: Bellantuono *et al.* (1980 ▶); Bartsch & Erker (1988 ▶); Baouid *et al.* (1994 ▶, 1996 ▶); Jalal *et al.* (2002 ▶); Rossi *et al.* (1960 ▶). Pour structures associées, voir: Chiaroni *et al.* (1995 ▶); El Hazazi *et al.* (2000 ▶).
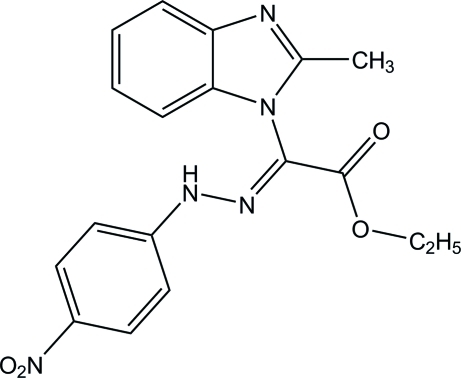


## Partie expérimentale

### 

#### Données cristallines

C_18_H_17_N_5_O_4_
*M* *_r_* = 367,37
Triclinique, 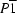

*a* = 8,269 (3) Å
*b* = 11,523 (2) Å
*c* = 19,853 (6) Å
α = 87,44 (2)°
β = 80,67 (3)°
γ = 76,80 (2)°
*V* = 1817,3 (9) Å^3^
*Z* = 4
Radiation Mo *K*α
μ = 0,10 mm^−1^
*T* = 300 K
0,4 × 0,18 × 0,12 mm

#### Collection des données

Diffractomètre Enraf–Nonius CAD-4
9121 réflexions mesurées
7912 réflexions indépendantes
4441 réflexions avec *I* > 2σ(*I*)
*R*_int_ = 0,018
2 réflexions de référence chaque 60 min diminution d’intensité: 1,0%

#### Affinement

*R*[*F* ^2^ > 2σ(*F* ^2^)] = 0,043
*wR*(*F* ^2^) = 0,131
*S* = 1,01
7912 réflexions
492 paramètres
Paramètres des atomes H contraints
Δρ_max_ = 0,17 e Å^−3^
Δρ_min_ = −0,19 e Å^−3^

### 

Collection des données: *CAD-4 EXPRESS* (Enraf–Nonius, 1989 ▶); affinement des paramètres de la maille: *CAD-4 EXPRESS*; réduction des données: *MolEN* (Fair, 1990 ▶); programme(s) pour la solution de la structure: *SHELXS97* (Sheldrick, 2008 ▶); programme(s) pour l’affinement de la structure: *SHELXL97* (Sheldrick, 2008 ▶); graphisme moléculaire: *ORTEP-3 for Windows* (Farrugia, 1997 ▶); logiciel utilisé pour préparer le matériel pour publication: *WinGX* (Farrugia, 1999 ▶).

## Supplementary Material

Crystal structure: contains datablock(s) I, global. DOI: 10.1107/S1600536811022148/is2730sup1.cif

Structure factors: contains datablock(s) I. DOI: 10.1107/S1600536811022148/is2730Isup2.hkl

Supplementary material file. DOI: 10.1107/S1600536811022148/is2730Isup3.cml

Additional supplementary materials:  crystallographic information; 3D view; checkCIF report

## Figures and Tables

**Table 1:** Géometrie des liaisons hydrogène (Å, °)

*D*—H⋯*A*	*D*—H	H⋯*A*	*D*⋯*A*	*D*—H⋯*A*
N2—HN2⋯N5^i^	0,86	2,19	2,954 (2)	148
N7—HN7⋯N10^ii^	0,86	2,19	2,967 (2)	150

Symmetry codes: (i) 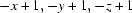; (ii) 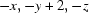.

**Fig. 1.**
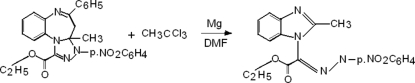
Schéma réactionnel du composé étudié.

**Fig. 2.**
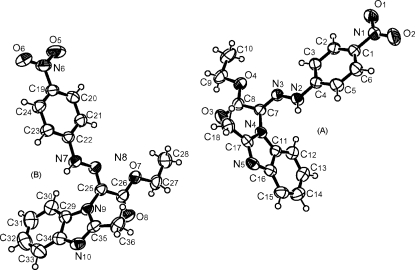
Représentation ORTEP de la structure avec les spheères d'atomes à 50% de probabilité.

## supplementary crystallographic information

### Comment

Les composés présentant un hétérocycle de différente taille accolé à un autre hétérocycle azoté à sept chaînons révélaient dans certains cas des propriétés pharmacologiques remarquables (Bellantuono *et al.*, 1980; Bartsch & Erker, 1988). Dans ce contexte, notre équipe de recherche s'est intéressée à la synthèse de nouvelles molécules dérivées des benzodiazépines et benzotriazépines possédant des structures homologues à des composés biologiquement actifs (Baouid *et al.*, 1996; El Hazazi *et al.*, 2000; Jalal *et al.*, 2002).

Développant ce travail, nous avons examiné le comportement de la [1,2,4]triazolo [4,3-*a*][1,5]benzodiazépine 1 (Baouid *et al.*, 1994) vis-à-vis de α,α,α-trichloroéthane avec le diméthylformamide (DMF) moyennant le Mg-Barbier. L'objectif de cette réaction est la formation des aziridines polysubstitués à partir de l'imine et le α,α,α-trichloroéthane. Alors un produit inattendu est obtenu (Fig. 1). La réaction est effectuée dans le DMF, à une température comprise entre 5°C et 15 °C. Opérant avec un équivalent de DMF et la solution de trichloroéthane en présence de Mg-Barbier, on obtient après agitation du mélange réactionnel pendant une journée, le produit 2 avec un rendement de 60%. Dans cette réaction le DMF joue le rôle du catalyseur et de solvant puisque aucune réaction n'a eu lieu dans le diéthyléther ou dans le tétrahydrofurane (THF) au lieu de DMF.

L'évolution de la réaction a été suivie par la chromatographie sous couche mince (CCM). La formation du composé 2 s'explique vraisemblablement par un réarrangement sigmatropique-1,3 du cycle à sept éléments (Rossi *et al.*, 1960), suivie de la perte du styrène aboutissant à un intermédiaire très instable qui se transpose à son tour pour conduire au produit réarrangé 2.

L'étude cristallographique a permis de déterminer clairement la stéréochimie du produit 2 (Fig. 2) et confirme ainsi le réarrangement du cycle à sept éléments suivi de la perte du styréne. La structure de ce composé, objet d'étude, cristallise dans le systéme triclinique, groupe d'espace P1, avec les paramètres de maille suivants: a = 8.269 (3) Å; b = 11.523 (2) Å; c = 19.853 (6) Å. L'unité asymétrique est formée de deux molécules cristallographiquement indépendantes A et B de formule identique: C_18_H_17_N_5_O_4_. Dans chaque molécule,le cycle 1–3,benzodiazole est pratiquement plan et présente un écart á la planéité de 0.0164 Å dans la molécule A et de 0.0098 Å dans B. Les caractéristiques géométriques de ce composé restent en bon accord avec celles observées dans d'autres composés simulaires (Chiaroni *et al.*, 1995; El Hazazi *et al.*, 2000).

### Experimental

La réaction est effectuée dans les conditions de Mg-Barbier en présence de DMF. Le magnésium (5,4 mmoles) est suspendu dans 40 ml de DMF dans un ballon tricol muni d'un thermomètre et d'une ampoule à brome, et refroidie à une température T < 5 °C. La moitié de la solution contenant le monocycloadduit 1 (0,54 mmoles), le α,α,α-trichloroéthane (0,65 mmoles) et DMF (5 ml) est ajouté rapidement au mélange réactionnel. Le reste du réactant est additionné durant 5 min, puis la réaction est agitée fortement et elle est suivie par chromatographie sur couche mince jusqu'à la consommation complète du monocycloadduit 1. Après traitement habituel du mélange réactionnel, le résidu obtenu est chromatographié sur colonne de gel de silice puis recristallisé dans l'acétate d'éthyle pour isoler le produit 2. Ce composé est obtenu avec un rendement de 60%. Solide beige; P.F. 201–202 °C.

### Refinement

Tous les atomes d'hydrogène ont été localisés par Fourier différence et affinés selon le modèle rigide avec C—H = 0.96 Å, *U*_iso_(H) = 1.2*U*_eq_(C) pour les atomes CH_3_, C—H = 0.97 Å, *U*_iso_(H) = 1.2*U*_eq_(C) pour les atomes CH_2_, C—H = 0.93 Å, *U*_iso_(H) = 1.2*U*_eq_(C) pour les atomes CH et N—H = 0.86 Å, *U*_iso_(H) = 1.2*U*_eq_(N) pour les atomes NH.

### Crystal data

**Table:**

C_18_H_17_N_5_O_4_	*Z* = 4
*M**_r_* = 367.37	*F*(000) = 768
Triclinic, *P*1	*D*_x_ = 1.343 Mg m^−^^3^
Hall symbol: -P 1	Mo *K*α radiation, λ = 0.71073 Å
*a* = 8.269 (3) Å	Cell parameters from 25 reflections
*b* = 11.523 (2) Å	θ = 10–15°
*c* = 19.853 (6) Å	µ = 0.10 mm^−^^1^
α = 87.44 (2)°	*T* = 300 K
β = 80.67 (3)°	Plate, yellow
γ = 76.80 (2)°	0.4 × 0.18 × 0.12 mm
*V* = 1817.3 (9) Å^3^	

### Data collection

**Table:**

Enraf–Nonius CAD-4 diffractometer	*R*_int_ = 0.018
Radiation source: fine-focus sealed tube	θ_max_ = 27.0°, θ_min_ = 1.8°
graphite	*h* = −10→10
ω/2θ scans	*k* = −1→14
9121 measured reflections	*l* = −25→25
7912 independent reflections	2 standard reflections every 60 min
4441 reflections with *I* > 2σ(*I*)	intensity decay: 1.0%

### Refinement

**Table:**

Refinement on *F*^2^	Primary atom site location: structure-invariant direct methods
Least-squares matrix: full	Secondary atom site location: difference Fourier map
*R*[*F*^2^ > 2σ(*F*^2^)] = 0.043	Hydrogen site location: difference Fourier map
*wR*(*F*^2^) = 0.131	H-atom parameters constrained
*S* = 1.01	*w* = 1/[σ^2^(*F*_o_^2^) + (0.0566*P*)^2^ + 0.2299*P*] where *P* = (*F*_o_^2^ + 2*F*_c_^2^)/3
7912 reflections	(Δ/σ)_max_ < 0.001
492 parameters	Δρ_max_ = 0.17 e Å^−^^3^
0 restraints	Δρ_min_ = −0.19 e Å^−^^3^

### Special details

**Table:**

Geometry. All e.s.d.'s (except the e.s.d. in the dihedral angle between two l.s. planes) are estimated using the full covariance matrix. The cell e.s.d.'s are taken into account individually in the estimation of e.s.d.'s in distances, angles and torsion angles; correlations between e.s.d.'s in cell parameters are only used when they are defined by crystal symmetry. An approximate (isotropic) treatment of cell e.s.d.'s is used for estimating e.s.d.'s involving l.s. planes.
Refinement. Refinement of *F*^2^ against ALL reflections. The weighted *R*-factor *wR* and goodness of fit *S* are based on *F*^2^, conventional *R*-factors *R* are based on *F*, with *F* set to zero for negative *F*^2^. The threshold expression of *F*^2^ > σ(*F*^2^) is used only for calculating *R*-factors(gt) *etc*. and is not relevant to the choice of reflections for refinement. *R*-factors based on *F*^2^ are statistically about twice as large as those based on *F*, and *R*-factors based on ALL data will be even larger.

### Fractional atomic coordinates and isotropic or equivalent isotropic displacement parameters (Å^2^)

**Table:**

	*x*	*y*	*z*	*U*_iso_*/*U*_eq_	
O1	1.1114 (2)	−0.22059 (13)	0.65166 (8)	0.0725 (4)	
O2	1.0965 (2)	−0.08283 (15)	0.72248 (9)	0.0889 (6)	
O3	0.3711 (2)	0.25412 (13)	0.32862 (8)	0.0678 (4)	
O4	0.54009 (18)	0.07952 (12)	0.35251 (7)	0.0590 (4)	
N1	1.0594 (2)	−0.11839 (15)	0.67144 (9)	0.0553 (4)	
N2	0.64051 (19)	0.20284 (12)	0.51891 (8)	0.0474 (4)	
HN2	0.6036	0.2757	0.5317	0.057*	
N3	0.59856 (19)	0.16436 (12)	0.46301 (8)	0.0444 (4)	
N4	0.41859 (18)	0.35473 (12)	0.44852 (7)	0.0415 (3)	
N5	0.33720 (19)	0.55351 (12)	0.44995 (8)	0.0486 (4)	
C1	0.9503 (2)	−0.03414 (16)	0.63150 (9)	0.0455 (4)	
C2	0.8921 (2)	−0.07698 (16)	0.57840 (9)	0.0498 (5)	
H2	0.9224	−0.1578	0.5683	0.060*	
C3	0.7888 (2)	0.00099 (15)	0.54058 (10)	0.0479 (4)	
H3	0.7481	−0.0270	0.5049	0.058*	
C4	0.7457 (2)	0.12177 (14)	0.55599 (9)	0.0418 (4)	
C5	0.8045 (3)	0.16319 (16)	0.60991 (10)	0.0541 (5)	
H5	0.7750	0.2440	0.6202	0.065*	
C6	0.9061 (3)	0.08517 (17)	0.64807 (11)	0.0557 (5)	
H6	0.9446	0.1124	0.6846	0.067*	
C7	0.5006 (2)	0.23631 (15)	0.42798 (9)	0.0426 (4)	
C8	0.4626 (2)	0.19271 (17)	0.36421 (10)	0.0478 (4)	
C9	0.5146 (3)	0.0271 (2)	0.29101 (11)	0.0681 (6)	
H9A	0.5655	0.0643	0.2508	0.082*	
H9B	0.3955	0.0378	0.2894	0.082*	
C10	0.5964 (3)	−0.1029 (2)	0.29373 (13)	0.0821 (8)	
H10A	0.5840	−0.1412	0.2535	0.098*	
H10B	0.5439	−0.1386	0.3334	0.098*	
H10C	0.7138	−0.1121	0.2960	0.098*	
C11	0.2933 (2)	0.38263 (15)	0.50564 (9)	0.0425 (4)	
C12	0.2219 (2)	0.31282 (17)	0.55511 (10)	0.0518 (5)	
H12	0.2577	0.2302	0.5555	0.062*	
C13	0.0942 (3)	0.3723 (2)	0.60408 (11)	0.0611 (5)	
H13	0.0434	0.3285	0.6382	0.073*	
C14	0.0407 (3)	0.4952 (2)	0.60341 (11)	0.0638 (6)	
H14	−0.0462	0.5321	0.6367	0.077*	
C15	0.1138 (3)	0.56380 (19)	0.55427 (11)	0.0581 (5)	
H15	0.0773	0.6464	0.5540	0.070*	
C16	0.2435 (2)	0.50651 (15)	0.50501 (10)	0.0449 (4)	
C17	0.4368 (2)	0.46180 (15)	0.41703 (9)	0.0433 (4)	
C18	0.5558 (3)	0.46803 (18)	0.35304 (10)	0.0565 (5)	
H18A	0.5017	0.4618	0.3145	0.068*	
H18B	0.6530	0.4037	0.3524	0.068*	
H18C	0.5896	0.5426	0.3509	0.068*	
O5	0.7356 (3)	0.28956 (17)	−0.17974 (11)	0.1057 (7)	
O6	0.7618 (2)	0.43406 (17)	−0.25022 (9)	0.0879 (5)	
O7	−0.25402 (19)	0.73598 (13)	0.15264 (8)	0.0698 (4)	
O8	−0.04397 (17)	0.57184 (12)	0.14022 (7)	0.0583 (4)	
N6	0.6970 (2)	0.39426 (18)	−0.19733 (11)	0.0673 (5)	
N7	0.1779 (2)	0.70257 (13)	−0.03046 (8)	0.0512 (4)	
HN7	0.1503	0.7765	−0.0418	0.061*	
N8	0.0946 (2)	0.65968 (13)	0.02521 (8)	0.0482 (4)	
N9	−0.10387 (19)	0.84754 (13)	0.04199 (8)	0.0466 (4)	
N10	−0.2019 (2)	1.04477 (14)	0.05012 (9)	0.0562 (4)	
C19	0.5655 (2)	0.47559 (17)	−0.15198 (10)	0.0507 (5)	
C20	0.4804 (3)	0.42932 (18)	−0.09549 (11)	0.0580 (5)	
H20	0.5092	0.3484	−0.0853	0.069 (6)*	
C21	0.3523 (3)	0.50385 (17)	−0.05419 (10)	0.0542 (5)	
H21	0.2941	0.4732	−0.0161	0.065*	
C22	0.3101 (2)	0.62482 (15)	−0.06951 (9)	0.0435 (4)	
C23	0.3984 (2)	0.66996 (17)	−0.12683 (10)	0.0532 (5)	
H23	0.3711	0.7510	−0.1370	0.064*	
C24	0.5258 (2)	0.59540 (18)	−0.16848 (10)	0.0544 (5)	
H24	0.5840	0.6252	−0.2069	0.065*	
C25	−0.0334 (2)	0.72821 (16)	0.06040 (9)	0.0468 (4)	
C26	−0.1234 (3)	0.68024 (17)	0.12275 (10)	0.0505 (5)	
C27	−0.1237 (3)	0.52064 (19)	0.20149 (10)	0.0628 (6)	
H27A	−0.2407	0.5242	0.1986	0.075*	
H27B	−0.1191	0.5646	0.2414	0.075*	
C28	−0.0312 (3)	0.3948 (2)	0.20676 (13)	0.0821 (7)	
H28A	0.0850	0.3923	0.2082	0.098*	
H28B	−0.0399	0.3515	0.1678	0.098*	
H28C	−0.0788	0.3593	0.2476	0.098*	
C29	−0.1922 (2)	0.88137 (17)	−0.01301 (10)	0.0495 (5)	
C30	−0.2235 (3)	0.8165 (2)	−0.06471 (11)	0.0635 (6)	
H30	−0.1833	0.7344	−0.0680	0.076*	
C31	−0.3176 (3)	0.8807 (3)	−0.11099 (13)	0.0794 (7)	
H31	−0.3396	0.8408	−0.1470	0.095*	
C32	−0.3802 (3)	1.0024 (3)	−0.10548 (15)	0.0858 (8)	
H32	−0.4443	1.0421	−0.1374	0.103*	
C33	−0.3497 (3)	1.0659 (2)	−0.05372 (14)	0.0732 (7)	
H33	−0.3930	1.1477	−0.0502	0.088*	
C34	−0.2524 (2)	1.00450 (18)	−0.00674 (11)	0.0547 (5)	
C35	−0.1178 (2)	0.95001 (17)	0.07824 (10)	0.0491 (5)	
C36	−0.0474 (3)	0.9487 (2)	0.14211 (11)	0.0652 (6)	
H36A	−0.0272	1.0258	0.1490	0.078*	
H36B	0.0565	0.8899	0.1390	0.078*	
H36C	−0.1257	0.9296	0.1798	0.078*	

### Atomic displacement parameters (Å^2^)

**Table:**

	*U*^11^	*U*^22^	*U*^33^	*U*^12^	*U*^13^	*U*^23^
O1	0.0922 (12)	0.0459 (9)	0.0725 (10)	0.0069 (8)	−0.0251 (9)	0.0030 (7)
O2	0.1183 (15)	0.0768 (11)	0.0719 (11)	0.0034 (10)	−0.0501 (10)	−0.0072 (9)
O3	0.0838 (11)	0.0588 (9)	0.0641 (9)	−0.0081 (8)	−0.0328 (8)	0.0002 (7)
O4	0.0719 (9)	0.0507 (8)	0.0512 (8)	−0.0031 (7)	−0.0112 (7)	−0.0159 (6)
N1	0.0613 (10)	0.0521 (10)	0.0494 (10)	−0.0047 (8)	−0.0121 (8)	0.0042 (8)
N2	0.0582 (10)	0.0301 (7)	0.0543 (9)	−0.0046 (7)	−0.0170 (8)	−0.0029 (7)
N3	0.0513 (9)	0.0366 (8)	0.0456 (8)	−0.0085 (7)	−0.0100 (7)	−0.0019 (7)
N4	0.0456 (8)	0.0331 (7)	0.0457 (8)	−0.0070 (6)	−0.0096 (7)	0.0008 (6)
N5	0.0521 (9)	0.0340 (8)	0.0625 (10)	−0.0073 (7)	−0.0216 (8)	0.0026 (7)
C1	0.0487 (10)	0.0400 (10)	0.0456 (10)	−0.0055 (8)	−0.0085 (8)	0.0039 (8)
C2	0.0616 (12)	0.0337 (9)	0.0503 (11)	−0.0034 (9)	−0.0076 (9)	−0.0020 (8)
C3	0.0607 (12)	0.0352 (9)	0.0486 (10)	−0.0071 (8)	−0.0155 (9)	−0.0023 (8)
C4	0.0445 (10)	0.0335 (9)	0.0472 (10)	−0.0086 (7)	−0.0076 (8)	0.0015 (7)
C5	0.0680 (13)	0.0328 (9)	0.0643 (13)	−0.0080 (9)	−0.0216 (10)	−0.0052 (9)
C6	0.0648 (13)	0.0459 (11)	0.0614 (13)	−0.0106 (10)	−0.0260 (10)	−0.0049 (9)
C7	0.0460 (10)	0.0353 (9)	0.0452 (10)	−0.0070 (8)	−0.0064 (8)	0.0005 (8)
C8	0.0528 (11)	0.0451 (11)	0.0455 (10)	−0.0109 (9)	−0.0076 (9)	0.0002 (8)
C9	0.0759 (15)	0.0747 (15)	0.0537 (13)	−0.0151 (12)	−0.0054 (11)	−0.0255 (11)
C10	0.103 (2)	0.0704 (16)	0.0693 (15)	−0.0232 (14)	0.0110 (14)	−0.0295 (13)
C11	0.0440 (10)	0.0400 (9)	0.0451 (10)	−0.0080 (8)	−0.0133 (8)	−0.0037 (8)
C12	0.0578 (12)	0.0464 (11)	0.0538 (12)	−0.0167 (9)	−0.0089 (9)	−0.0021 (9)
C13	0.0585 (13)	0.0740 (15)	0.0546 (12)	−0.0248 (11)	−0.0043 (10)	−0.0063 (11)
C14	0.0476 (12)	0.0792 (16)	0.0636 (14)	−0.0096 (11)	−0.0062 (10)	−0.0228 (12)
C15	0.0527 (12)	0.0503 (12)	0.0711 (14)	−0.0013 (9)	−0.0198 (11)	−0.0152 (10)
C16	0.0443 (10)	0.0391 (10)	0.0541 (11)	−0.0071 (8)	−0.0174 (9)	−0.0060 (8)
C17	0.0478 (10)	0.0353 (9)	0.0517 (11)	−0.0106 (8)	−0.0213 (9)	0.0054 (8)
C18	0.0609 (13)	0.0556 (12)	0.0571 (12)	−0.0196 (10)	−0.0153 (10)	0.0115 (10)
O5	0.1034 (15)	0.0657 (12)	0.1168 (16)	0.0254 (10)	0.0128 (12)	−0.0075 (11)
O6	0.0697 (11)	0.1044 (14)	0.0695 (11)	0.0060 (10)	0.0141 (9)	−0.0099 (10)
O7	0.0714 (10)	0.0590 (9)	0.0667 (10)	−0.0101 (8)	0.0210 (8)	−0.0078 (7)
O8	0.0604 (9)	0.0589 (9)	0.0490 (8)	−0.0115 (7)	0.0058 (6)	0.0084 (6)
N6	0.0516 (11)	0.0700 (13)	0.0706 (13)	0.0063 (9)	−0.0063 (9)	−0.0125 (10)
N7	0.0593 (10)	0.0367 (8)	0.0506 (9)	−0.0082 (7)	0.0080 (8)	0.0000 (7)
N8	0.0550 (9)	0.0439 (9)	0.0438 (9)	−0.0130 (7)	0.0013 (7)	−0.0032 (7)
N9	0.0535 (9)	0.0387 (8)	0.0454 (9)	−0.0110 (7)	0.0008 (7)	−0.0062 (7)
N10	0.0554 (10)	0.0417 (9)	0.0668 (11)	−0.0118 (8)	0.0070 (8)	−0.0069 (8)
C19	0.0433 (10)	0.0530 (11)	0.0510 (11)	−0.0007 (9)	−0.0054 (9)	−0.0089 (9)
C20	0.0653 (13)	0.0416 (11)	0.0602 (13)	0.0019 (10)	−0.0108 (11)	0.0016 (9)
C21	0.0626 (13)	0.0472 (11)	0.0490 (11)	−0.0103 (9)	−0.0011 (9)	0.0029 (9)
C22	0.0460 (10)	0.0383 (9)	0.0447 (10)	−0.0080 (8)	−0.0037 (8)	−0.0034 (8)
C23	0.0557 (12)	0.0406 (10)	0.0583 (12)	−0.0108 (9)	0.0057 (9)	0.0011 (9)
C24	0.0501 (11)	0.0577 (12)	0.0520 (11)	−0.0130 (9)	0.0040 (9)	−0.0022 (9)
C25	0.0539 (11)	0.0405 (10)	0.0447 (10)	−0.0119 (9)	−0.0011 (9)	−0.0045 (8)
C26	0.0592 (12)	0.0468 (11)	0.0448 (11)	−0.0154 (9)	0.0009 (9)	−0.0065 (9)
C27	0.0709 (14)	0.0708 (14)	0.0454 (11)	−0.0218 (12)	0.0005 (10)	0.0091 (10)
C28	0.0934 (19)	0.0759 (17)	0.0727 (16)	−0.0212 (14)	−0.0039 (14)	0.0217 (13)
C29	0.0490 (11)	0.0492 (11)	0.0506 (11)	−0.0150 (9)	−0.0033 (9)	0.0013 (9)
C30	0.0691 (14)	0.0624 (13)	0.0635 (13)	−0.0243 (11)	−0.0095 (11)	−0.0038 (11)
C31	0.0830 (18)	0.096 (2)	0.0718 (16)	−0.0378 (16)	−0.0268 (14)	0.0076 (14)
C32	0.0745 (17)	0.101 (2)	0.091 (2)	−0.0273 (16)	−0.0336 (15)	0.0261 (17)
C33	0.0617 (14)	0.0619 (14)	0.0919 (18)	−0.0097 (11)	−0.0118 (13)	0.0203 (13)
C34	0.0482 (11)	0.0489 (11)	0.0655 (13)	−0.0144 (9)	−0.0007 (10)	0.0026 (10)
C35	0.0497 (11)	0.0435 (11)	0.0515 (11)	−0.0152 (9)	0.0084 (9)	−0.0085 (8)
C36	0.0787 (15)	0.0666 (14)	0.0546 (13)	−0.0289 (12)	−0.0007 (11)	−0.0142 (10)

### Geometric parameters (Å, °)

**Table:**

O1—N1	1.217 (2)	O5—N6	1.226 (2)
O2—N1	1.218 (2)	O6—N6	1.220 (2)
O3—C8	1.207 (2)	O7—C26	1.200 (2)
O4—C8	1.328 (2)	O8—C26	1.331 (2)
O4—C9	1.452 (2)	O8—C27	1.455 (2)
N1—C1	1.466 (2)	N6—C19	1.471 (3)
N2—N3	1.337 (2)	N7—N8	1.342 (2)
N2—C4	1.395 (2)	N7—C22	1.392 (2)
N2—HN2	0.8600	N7—HN7	0.8600
N3—C7	1.285 (2)	N8—C25	1.288 (2)
N4—C17	1.386 (2)	N9—C35	1.383 (2)
N4—C11	1.401 (2)	N9—C29	1.403 (2)
N4—C7	1.423 (2)	N9—C25	1.422 (2)
N5—C17	1.309 (2)	N10—C35	1.309 (3)
N5—C16	1.397 (2)	N10—C34	1.397 (3)
C1—C6	1.380 (3)	C19—C20	1.378 (3)
C1—C2	1.381 (3)	C19—C24	1.382 (3)
C2—C3	1.378 (3)	C20—C21	1.377 (3)
C2—H2	0.9300	C20—H20	0.9300
C3—C4	1.391 (2)	C21—C22	1.390 (3)
C3—H3	0.9300	C21—H21	0.9300
C4—C5	1.390 (3)	C22—C23	1.397 (3)
C5—C6	1.374 (3)	C23—C24	1.378 (3)
C5—H5	0.9300	C23—H23	0.9300
C6—H6	0.9300	C24—H24	0.9300
C7—C8	1.487 (3)	C25—C26	1.490 (3)
C9—C10	1.498 (3)	C27—C28	1.485 (3)
C9—H9A	0.9700	C27—H27A	0.9700
C9—H9B	0.9700	C27—H27B	0.9700
C10—H10A	0.9600	C28—H28A	0.9600
C10—H10B	0.9600	C28—H28B	0.9600
C10—H10C	0.9600	C28—H28C	0.9600
C11—C12	1.385 (3)	C29—C30	1.387 (3)
C11—C16	1.393 (2)	C29—C34	1.396 (3)
C12—C13	1.388 (3)	C30—C31	1.380 (3)
C12—H12	0.9300	C30—H30	0.9300
C13—C14	1.384 (3)	C31—C32	1.382 (4)
C13—H13	0.9300	C31—H31	0.9300
C14—C15	1.379 (3)	C32—C33	1.375 (4)
C14—H14	0.9300	C32—H32	0.9300
C15—C16	1.392 (3)	C33—C34	1.394 (3)
C15—H15	0.9300	C33—H33	0.9300
C17—C18	1.485 (3)	C35—C36	1.477 (3)
C18—H18A	0.9600	C36—H36A	0.9600
C18—H18B	0.9600	C36—H36B	0.9600
C18—H18C	0.9600	C36—H36C	0.9600
			
C8—O4—C9	116.83 (16)	C26—O8—C27	115.74 (16)
O1—N1—O2	122.68 (18)	O6—N6—O5	123.4 (2)
O1—N1—C1	118.63 (17)	O6—N6—C19	118.66 (19)
O2—N1—C1	118.68 (17)	O5—N6—C19	117.9 (2)
N3—N2—C4	118.31 (14)	N8—N7—C22	118.60 (15)
N3—N2—HN2	120.8	N8—N7—HN7	120.7
C4—N2—HN2	120.8	C22—N7—HN7	120.7
C7—N3—N2	119.99 (15)	C25—N8—N7	119.93 (16)
C17—N4—C11	106.86 (14)	C35—N9—C29	106.74 (15)
C17—N4—C7	129.14 (15)	C35—N9—C25	127.53 (16)
C11—N4—C7	123.94 (15)	C29—N9—C25	125.29 (15)
C17—N5—C16	105.97 (14)	C35—N10—C34	106.03 (16)
C6—C1—C2	121.48 (17)	C20—C19—C24	121.67 (18)
C6—C1—N1	119.75 (17)	C20—C19—N6	118.98 (19)
C2—C1—N1	118.76 (16)	C24—C19—N6	119.29 (19)
C3—C2—C1	119.47 (17)	C21—C20—C19	119.52 (18)
C3—C2—H2	120.3	C21—C20—H20	120.2
C1—C2—H2	120.3	C19—C20—H20	120.2
C2—C3—C4	119.62 (17)	C20—C21—C22	120.00 (19)
C2—C3—H3	120.2	C20—C21—H21	120.0
C4—C3—H3	120.2	C22—C21—H21	120.0
C5—C4—C3	120.12 (17)	C21—C22—N7	121.86 (17)
C5—C4—N2	118.87 (16)	C21—C22—C23	119.56 (17)
C3—C4—N2	120.99 (16)	N7—C22—C23	118.55 (16)
C6—C5—C4	120.21 (17)	C24—C23—C22	120.53 (18)
C6—C5—H5	119.9	C24—C23—H23	119.7
C4—C5—H5	119.9	C22—C23—H23	119.7
C5—C6—C1	119.08 (18)	C23—C24—C19	118.72 (18)
C5—C6—H6	120.5	C23—C24—H24	120.6
C1—C6—H6	120.5	C19—C24—H24	120.6
N3—C7—N4	123.59 (16)	N8—C25—N9	124.46 (17)
N3—C7—C8	118.85 (16)	N8—C25—C26	119.50 (17)
N4—C7—C8	117.48 (15)	N9—C25—C26	115.93 (16)
O3—C8—O4	125.28 (18)	O7—C26—O8	125.24 (18)
O3—C8—C7	123.09 (17)	O7—C26—C25	121.83 (19)
O4—C8—C7	111.63 (16)	O8—C26—C25	112.93 (17)
O4—C9—C10	106.55 (19)	O8—C27—C28	107.56 (18)
O4—C9—H9A	110.4	O8—C27—H27A	110.2
C10—C9—H9A	110.4	C28—C27—H27A	110.2
O4—C9—H9B	110.4	O8—C27—H27B	110.2
C10—C9—H9B	110.4	C28—C27—H27B	110.2
H9A—C9—H9B	108.6	H27A—C27—H27B	108.5
C9—C10—H10A	109.5	C27—C28—H28A	109.5
C9—C10—H10B	109.5	C27—C28—H28B	109.5
H10A—C10—H10B	109.5	H28A—C28—H28B	109.5
C9—C10—H10C	109.5	C27—C28—H28C	109.5
H10A—C10—H10C	109.5	H28A—C28—H28C	109.5
H10B—C10—H10C	109.5	H28B—C28—H28C	109.5
C12—C11—C16	122.47 (17)	C30—C29—C34	122.8 (2)
C12—C11—N4	132.62 (16)	C30—C29—N9	132.18 (18)
C16—C11—N4	104.91 (16)	C34—C29—N9	104.98 (17)
C11—C12—C13	116.67 (19)	C31—C30—C29	116.2 (2)
C11—C12—H12	121.7	C31—C30—H30	121.9
C13—C12—H12	121.7	C29—C30—H30	121.9
C14—C13—C12	121.7 (2)	C30—C31—C32	122.1 (2)
C14—C13—H13	119.2	C30—C31—H31	119.0
C12—C13—H13	119.2	C32—C31—H31	119.0
C15—C14—C13	121.2 (2)	C33—C32—C31	121.4 (2)
C15—C14—H14	119.4	C33—C32—H32	119.3
C13—C14—H14	119.4	C31—C32—H32	119.3
C14—C15—C16	118.37 (19)	C32—C33—C34	118.3 (2)
C14—C15—H15	120.8	C32—C33—H33	120.9
C16—C15—H15	120.8	C34—C33—H33	120.9
C15—C16—C11	119.65 (19)	C33—C34—C29	119.3 (2)
C15—C16—N5	130.16 (18)	C33—C34—N10	130.8 (2)
C11—C16—N5	110.17 (16)	C29—C34—N10	109.93 (18)
N5—C17—N4	112.06 (17)	N10—C35—N9	112.28 (18)
N5—C17—C18	125.37 (16)	N10—C35—C36	125.24 (18)
N4—C17—C18	122.57 (16)	N9—C35—C36	122.47 (18)
C17—C18—H18A	109.5	C35—C36—H36A	109.5
C17—C18—H18B	109.5	C35—C36—H36B	109.5
H18A—C18—H18B	109.5	H36A—C36—H36B	109.5
C17—C18—H18C	109.5	C35—C36—H36C	109.5
H18A—C18—H18C	109.5	H36A—C36—H36C	109.5
H18B—C18—H18C	109.5	H36B—C36—H36C	109.5
			
C4—N2—N3—C7	179.34 (16)	C22—N7—N8—C25	−176.61 (17)
O1—N1—C1—C6	−172.27 (19)	O6—N6—C19—C20	173.9 (2)
O2—N1—C1—C6	6.6 (3)	O5—N6—C19—C20	−6.2 (3)
O1—N1—C1—C2	8.6 (3)	O6—N6—C19—C24	−3.6 (3)
O2—N1—C1—C2	−172.50 (19)	O5—N6—C19—C24	176.4 (2)
C6—C1—C2—C3	0.6 (3)	C24—C19—C20—C21	0.0 (3)
N1—C1—C2—C3	179.68 (17)	N6—C19—C20—C21	−177.33 (19)
C1—C2—C3—C4	0.5 (3)	C19—C20—C21—C22	−0.2 (3)
C2—C3—C4—C5	−1.0 (3)	C20—C21—C22—N7	177.85 (19)
C2—C3—C4—N2	−179.60 (17)	C20—C21—C22—C23	−0.1 (3)
N3—N2—C4—C5	173.32 (16)	N8—N7—C22—C21	3.0 (3)
N3—N2—C4—C3	−8.1 (3)	N8—N7—C22—C23	−178.99 (17)
C3—C4—C5—C6	0.3 (3)	C21—C22—C23—C24	0.6 (3)
N2—C4—C5—C6	178.94 (18)	N7—C22—C23—C24	−177.45 (19)
C4—C5—C6—C1	0.8 (3)	C22—C23—C24—C19	−0.7 (3)
C2—C1—C6—C5	−1.3 (3)	C20—C19—C24—C23	0.4 (3)
N1—C1—C6—C5	179.64 (18)	N6—C19—C24—C23	177.78 (19)
N2—N3—C7—N4	−6.2 (3)	N7—N8—C25—N9	4.8 (3)
N2—N3—C7—C8	177.09 (15)	N7—N8—C25—C26	−179.04 (17)
C17—N4—C7—N3	116.7 (2)	C35—N9—C25—N8	−118.9 (2)
C11—N4—C7—N3	−66.5 (2)	C29—N9—C25—N8	69.7 (3)
C17—N4—C7—C8	−66.6 (2)	C35—N9—C25—C26	64.9 (2)
C11—N4—C7—C8	110.17 (19)	C29—N9—C25—C26	−106.5 (2)
C9—O4—C8—O3	1.4 (3)	C27—O8—C26—O7	−1.3 (3)
C9—O4—C8—C7	−179.17 (16)	C27—O8—C26—C25	178.91 (16)
N3—C7—C8—O3	179.47 (19)	N8—C25—C26—O7	−171.24 (19)
N4—C7—C8—O3	2.6 (3)	N9—C25—C26—O7	5.2 (3)
N3—C7—C8—O4	0.0 (2)	N8—C25—C26—O8	8.5 (3)
N4—C7—C8—O4	−176.83 (15)	N9—C25—C26—O8	−175.03 (15)
C8—O4—C9—C10	−173.35 (18)	C26—O8—C27—C28	172.01 (18)
C17—N4—C11—C12	179.24 (19)	C35—N9—C29—C30	−178.4 (2)
C7—N4—C11—C12	1.9 (3)	C25—N9—C29—C30	−5.6 (3)
C17—N4—C11—C16	−0.09 (18)	C35—N9—C29—C34	0.89 (19)
C7—N4—C11—C16	−177.45 (15)	C25—N9—C29—C34	173.76 (17)
C16—C11—C12—C13	1.2 (3)	C34—C29—C30—C31	0.5 (3)
N4—C11—C12—C13	−178.02 (18)	N9—C29—C30—C31	179.8 (2)
C11—C12—C13—C14	0.2 (3)	C29—C30—C31—C32	−1.3 (4)
C12—C13—C14—C15	−0.8 (3)	C30—C31—C32—C33	0.8 (4)
C13—C14—C15—C16	0.0 (3)	C31—C32—C33—C34	0.5 (4)
C14—C15—C16—C11	1.4 (3)	C32—C33—C34—C29	−1.2 (3)
C14—C15—C16—N5	179.41 (18)	C32—C33—C34—N10	−179.9 (2)
C12—C11—C16—C15	−2.0 (3)	C30—C29—C34—C33	0.7 (3)
N4—C11—C16—C15	177.39 (16)	N9—C29—C34—C33	−178.74 (18)
C12—C11—C16—N5	179.57 (16)	C30—C29—C34—N10	179.64 (18)
N4—C11—C16—N5	−1.01 (19)	N9—C29—C34—N10	0.2 (2)
C17—N5—C16—C15	−176.41 (19)	C35—N10—C34—C33	177.5 (2)
C17—N5—C16—C11	1.77 (19)	C35—N10—C34—C29	−1.3 (2)
C16—N5—C17—N4	−1.85 (19)	C34—N10—C35—N9	1.9 (2)
C16—N5—C17—C18	177.58 (17)	C34—N10—C35—C36	−176.71 (18)
C11—N4—C17—N5	1.26 (19)	C29—N9—C35—N10	−1.8 (2)
C7—N4—C17—N5	178.44 (16)	C25—N9—C35—N10	−174.49 (17)
C11—N4—C17—C18	−178.19 (16)	C29—N9—C35—C36	176.86 (17)
C7—N4—C17—C18	−1.0 (3)	C25—N9—C35—C36	4.2 (3)

### Hydrogen-bond geometry (Å, °)

**Table:**

*D*—H···*A*	*D*—H	H···*A*	*D*···*A*	*D*—H···*A*
N2—HN2···N5^i^	0.86	2.19	2.954 (2)	148.
N7—HN7···N10^ii^	0.86	2.19	2.967 (2)	150.

Symmetry codes: (i) −*x*+1, −*y*+1, −*z*+1; (ii) −*x*, −*y*+2, −*z*.
